# Clinical characteristics of patients with *SALL1*-related disorder

**DOI:** 10.1007/s00467-025-06878-z

**Published:** 2025-07-14

**Authors:** Yoshitaka Asagai, Yu Tanaka, Hiroaki Hanafusa, China Nagano, Tomoko Horinouchi, Shingo Ishimori, Hiroshi Kaito, Kazumoto Iijima, Kandai Nozu, Naoya Morisada

**Affiliations:** 1 Department of Pediatrics, Kobe University Graduate School of Medicine, Kobe, Hyogo Japan; 2 Department of Clinical Genetics, Hyogo Prefectural Kobe Children’s Hospita, Kobe, Hyogo Japan; 3 Department of Nephrology, Hyogo Prefectural Kobe Children’s Hospital, Kobe, Hyogo Japan

**Keywords:** *SALL1*, Townes-Brocks syndrome 1, Branchio-oto-renal syndrome, CAKUT, Genetic analysis

## Abstract

**Background:**

The *Spalt-like transcription factor 1* (*SALL1)* gene is essential for kidney development. Pathogenic *SALL1* variants cause Townes-Brocks syndrome 1 (TBS1), which typically presents with imperforate anus, dysplastic ears, and digital anomalies. However, clinical features vary widely. Some patients present only with dysplastic ears and hearing loss (HL) or with congenital anomalies of the kidney and urinary tract (CAKUT), resembling branchio-oto-renal syndrome (BORS), a presentation referred to as Townes-Brocks branchio-oto-renal-like (TBS BOR-like) syndrome. In this study, we aimed to describe the clinical characteristics of patients with *SALL1*-related disorders in the Japanese population.

**Methods:**

We analyzed phenotypes of a nationwide cohort comprising 1108 families with chronic kidney disease (CKD) or mild urinary anomalies, using genetic testing conducted from 2010 to 2024.

**Results:**

We identified *SALL1* variants in 14 families (20 individuals): seven frameshift, four nonsense, one missense, one exon 2 deletion, and one whole-gene deletion. Ten variants were novel. The median age at diagnosis was 16 years (male:female = 13:7). Dysplastic ears were observed in 45%, HL in 40%, digital anomalies in 40%, and anorectal malformations in 25%. Based on clinical features, eight individuals were diagnosed with TBS1, four with TBS BOR-like syndrome, and seven with non-syndromic CAKUT. One case lacked detailed clinical data. Most variants were truncating and located in exon 2.

**Conclusions:**

*SALL1*-related disorders exhibit broad phenotypic variability. Some cases present with atypical features overlapping with TBS BOR-like syndrome or isolated CAKUT, rather than with typical TBS1. These findings enhance the understanding and diagnosis of *SALL1*-related disorders.

**Graphical abstract:**

**Figure:**
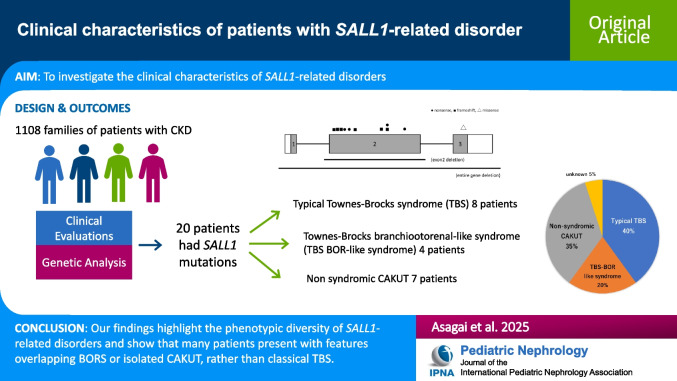
A higher resolution version of the Graphical abstract is available as Supplementary information

**Supplementary Information:**

The online version contains supplementary material available at 10.1007/s00467-025-06878-z.

## Introduction

The *Spalt-like transcription factor 1 (SALL1)* gene, located on chromosome 16q12.1, encodes a zinc-finger repressor transcription factor. *SALL1* is critical for normal kidney development; biallelic loss of function results in severe kidney dysgenesis, whereas monoallelic pathogenic variants can allow partial kidney development [1]. *SALL1* is the causative gene of Townes-Brocks syndrome 1 (TBS1, OMIM#107,480) [2, 3]. TBS1 is a rare autosomal dominant disorder characterized by a triad of imperforate anus or anal atresia, dysplastic ears, and digital malformations [4]. In addition, kidney diseases, including congenital anomalies of the kidney and urinary tract (CAKUT) or focal segmental glomerulosclerosis—congenital heart disease (CHD), hearing loss (HL), and ocular involvement—may also be part of the TBS1 clinical spectrum [4–6]. The penetrance of *SALL1* variants is considered high; however, the clinical phenotypes vary widely, even within the same family [4]. Some patients present with dysplastic ears, HL, and CAKUT in the absence of digital or anal anomalies, leading to a diagnosis of branchio-oto-renal (BOR) syndrome [7, 8]; this presentation is referred to as Townes-Brocks branchio-oto-renal-like (TBS BOR-like) syndrome [7]. Furthermore, a rare case of a patient with *SALL1* variant presenting with only bilateral renal hypodysplasia (RHD) has been reported [9]. Consequently, comprehensive genetic analysis using next-generation sequencing (NGS) can identify *SALL1* variants in patients with non-syndromic CAKUT or chronic kidney disease (CKD) of unknown etiology.

In this study, we aimed to describe the clinical characteristics of patients with *SALL1*-related disorders in the Japanese population.

## Materials and methods

### Ethical considerations

All procedures involving human participants adhered to the ethical standards of the Institutional Review Board of Kobe University Graduate School of Medicine (approval numbers 65 and 301), as well as the 1964 Helsinki Declaration and its later amendments or comparable ethical standards. Informed consent was obtained from all patients or their parents.

### Patients

We performed genetic testing on 1108 families of patients with CKD of unknown etiology between 2010 and November 2024. These patients, from across Japan, exhibited minimal urinary abnormalities. A subset exhibited clinical features suggestive of specific syndromes such as TBS or BORS, including CAKUT, nephronophthisis, polycystic kidney disease, and autosomal dominant tubulointerstitial kidney disease.

### Genetic analysis

Deoxyribonucleic acid (DNA) was isolated from peripheral blood samples using the QuickGene Mini 80 system (Wako Pure Chemical Industries, Ltd., Tokyo, Japan), following the manufacturer’s instructions. We conducted direct sequencing, multiplex ligation-dependent probe amplification (MLPA), array comparative genomic hybridization (aCGH), and targeted sequencing of genes associated with inherited disease using NGS. Direct sequencing was performed as previously described [10]. MLPA was performed using SALSA P180 (MRC-Holland, Amsterdam, Netherlands), and aCGH was carried out with the SurePrint G3 Human CGH Microarray 60 K kit (Agilent Technologies, Santa Clara, CA, USA), following the manufacturer’s instructions.

For NGS, we used HaloPlex or SureSelect (Agilent Technologies, Santa Clara, CA, USA) following the manufacturers’ instructions, and sequencing was performed using the MiSeq platform (Illumina). HaloPlex and SureSelect facilitated targeted sequencing of 172 (version 4, Supplementary Table 1), 164 (version 6, Supplementary Table 2), 181 (version 7, Supplementary Table 3), 181 (version 8, Supplementary Table 4), 193 (version 9, Supplementary Table 5), and 188 genes (version 10, Supplementary Table 6) associated with CAKUT or cystic kidney disease, as catalogued in the OMIM (http://www.omim.org/) and PubMed (http://www.ncbi.nlm.nih.gov/pubmed) databases. We analyzed data using SureCall 4.0 (Agilent Technologies), end-to-end NGS data analysis software. The complementary DNA reference sequence for *SALL1* used in this study was NM_002968.3. Variant pathogenicity was predicted according to American College of Medical Genetics (ACMG) guidelines [11], using multiple databases and tools, including Combined Annotation Dependent Depletion (CADD, https://cadd.gs.washington.edu/), Protein Variation Effect Analyzer (http://provean.jcvi.org/index.php), Sorting Intolerant From Tolerant (https://sift.bii.a-star.edu.sg/), Polymorphism Phenotyping v2 (http://genetics.bwh.harvard.edu/pph2/), and MutationTaster (https://www.genecascade.org/MutationTaster2025/).

### Clinical descriptions

Generally, when a heterozygous pathogenic variant in the *SALL1* gene is identified in patients with TBS-associated features, including imperforate anus or anal atresia, dysplastic ears, and digital malformations, the diagnosis is TBS1 [4]. In this study, “typical TBS” was defined as cases presenting with the cardinal features of imperforate anus or anal stenosis, dysplastic ears, and characteristic thumb malformations, which are major diagnostic features of TBS1. “TBS BOR-like syndrome” was defined as cases of *SALL1*-related TBS that lacked the cardinal features of typical TBS but presented with external ear abnormalities and/or branchial abnormalities, including HL. “Non-syndromic CAKUT” was defined as disease of the kidney and urinary tract without other specific manifestations.

## Results

### Patient characteristics

We identified 20 patients from 14 of 1108 families with CKD and minimal urinary abnormalities (Table 1). The median age of the patients at the last follow-up was 16 years. The male-to-female ratio was 13:7. Five patients had a family history of typical TBS or CKD (Table 2).

**Table 1:** Clinical and genetic information of the patients in the study

	Pt	Age at last follow-up	Gender	FH	Kidney disease	eGFR (ml/min/1.73 m2)eGFR (ml/min/1.73 m2)	CKD stage	HL	HL type	DE	Ocular	ID	Digital anomalies	Imperforte anus
1	SC140	6	M	+	RHD	NA	3	+	Sensory	+	-	Mild	r 3–4 syndactyly	Atresia of anus
	Father	44	M	CKD	60.0	2	+	NA	-	-	-	-	-	
2	SC327	31	M	+	RHD	26.6	4	-	-	-	-	-	Preaxillar polydactyly	-
	Mother	NA	F	NA	NA	-	-	-	-	-	-	-	-	
3	SC334	19	M	-	CKD	59.1	3	+	-	+	-	-	-	-
4	SC384	3	M	-	RHD, r Cystic kidney	67.0	2	+	NA	+	-	-	r preaxillar polydactyly, b triphalangiar preaxillar polydactyly, b triphalangia	Atresia of anus
5	SC464	16	M	-	CKD	47.6	3	+	NA	-	-	-	b preaxillar polydactyly	Atresia of anus
6	SC505	3	M	-	Ureterocele, l double renal pelvis and ureter,	94.0	1	-	-	-	-	-	-	-
7	SC554	8	F	+	CKD	70.5	2	-	-	-	-	-	-	-
	Father	34	M	CKD	on HD	5	-	-	-	-	-	-	-	
	Sister	6	F		CKD	NA	2	-	-	-	-	-	-	-
8	SC587	20	M	+	RHD	50.0	3	-	-	-	-	-	l. thumb syndactyly	Anal stenosis
	Brother	25	M	CKD	56.0	3	-	-	-	-	-	-	-	
9	SC624	10	F	-	RHD	38.8	3	+	NA	+	-	-	-	-
10	SC652	18	M	+	RHD	32.0	3	+	Mixed	+	Heterotropia, l amblyopiaheterotropia, l amblyopia	-	b duplicate thumb	-
	Brother	11	M	-	-	1	-	-	+	-	-	r duplicate thumb	-	
11	SC693	15	F	-	CKD	67.0	2	+	NA	+	ptosis	-	-	-
12	SC810	26	F	-	CKD	46.5	3	-	-	-	-	-	-	-
13	SC903	2	F	-	CKD	31.3	3	-	-	+	-	-	-	-
14	SC1136	27	M	-	CKD	40.0	3	+	NA	+	-	+	b thumb and pinky flexion	+

	CHD	Others	Clinical diagnosis	cDNA	Amino acids	HGMD	dbSNP	De novo	ACMG pathogenicity	Method	NGS Version	Inheritance	Ref
1	VSD	Growth failure	Typical TBS	rsa 16q12.1(SALL1exons2) × 1	-	-		N		MLPA	-	Paternal	
	-	-	Non-syndromic CAKUT					NA		MLPA	-	NA	
2	-	-	Typical TBS	c.3154C > T	p.(Gln1052*)	-	rs1057518131 (CLIN_likely_pathogenic)	N	Pathogenic (PVS1, PM2, PP3, PP4, PP5)	NGS	4	Maternal	
	-	-	-					NA		Sanger	-	NA	
3	-	-	TBS BOR-like syndrome	c.1295dup	p.(Asn432Lysfs*6)	CI186007	-	NA	Pathogenic (PVS1, PM2, PP3, PP4)	NGS	4	unknown	8
4	-	Hypothyroidism	Typical TBS	c.1146-1149del	p.(Leu383*)	-	-	Y	Pathogenic (PVS1, PM2, PP3, PP4)	Sanger	-	De novo	
5	-	-	Typical TBS	c.1031 T > A	p.(Leu344*)	-	-	Y	Pathogenic (PVS1, PM2, PP3, PP4)	Sanger	-	De novo	
6	-	-	Non-syndromic CAKUT	c.3956A > T	p.(Lys1319Met)	-	-	NA	VUS (PM1, PM2, PP4, BP1)	NGS	6	Unknown	
7	-	-	Non-syndromic CAKUT	c.2775del	p.(Met926Cysfs*40)	-	-	N	Pathogenic (PVS1, PM2, PP3, PP4)	NGS	7	Paternal	
	-	-	Non-syndromic CAKUT					NA		Sanger	-	NA	
	-	-	Non-syndromic CAKUT					N		Sanger	-	Paternal	
8	-	-	Typical TBS	c.2928del	p.(His976Glnfs*69)	-	-	NA	Pathogenic (PVS1, PM2, PP3, PP4)	NGS	7	Paternal?	
	-	-	Non-syndromic CAKUT				-	NA		Sanger			
9	-	Hypothyroidism	TBS BOR-like syndrome	c.953dup	p.(Ile319Asnfs*36)	-	-	Y	Pathogenic (PVS1, PM2, PP3, PP4)	NGS	8	De novo	
10	-	-	Typical TBS	c.2845C > T	p.(Gln949*)	-	-	NA	Pathogenic (PVS1, PM2, PP3, PP4)	Sanger	-	Paternal?	
	-	-	Typical TBS					NA		Sanger	-	Paternal?	
11	-	Short stature, ventricular enlargement	TBS BOR-like syndrome	arr[GRCh37] 16q12.1(50870773_51439346) × 1	-	-		NA		aCGH	-	NA	
12	-	-	non-syndromic CAKUT	c.203del	p.(Asn68Ilefs*3)	-	-	NA	Pathogenic (PVS1, PM2, PP3, PP4)	NGS	9	NA	
13	-	-	TBS BOR-like syndrome	c.2845_2846del	p.(Gln949Glufs*26)	-	-	NA	Pathogenic (PVS1, PM2, PP3, PP4)	NGS	10	NA	
14	-	-	Typical TBS	c.845del	p.(Leu282Cysfs*4)	-	-	NA	Pathogenic (PVS1, PM2, PP3, PP4)	Sanger	-	NA	

*aCGH*, array comparative genomic hybridization;* b*, bilateral; *BOR*, branchiootorenal; *CAKUT*, congenital anomalies of the kidney and urinary tract; *CHD*, congenital heart disease; *CKD*, chronic kidney disease; *DE*, dysplastic ears; *eGFR*; estimated glomerular filtration rate; *F*, female; *FH*, family history; *HD*, hemodialysis; *ID*, intellectual disability;* l*, left; *M*, male; *MLPA*, multiple ligation-dependent probe amplification;* N*, no; *NA*, not available; *NGS*, next generation sequencing; *Pt,* patient; *r*, right; *Ref*, references;* RHD, *renal hypodysplasia*; TBS*, Townes-Brocks syndrome; *VSD*, ventricular septal defect; *Y*, yes

**Table 2:** Summary of clinical information in the study

**Clinical manifestations**	
Number of patients (families)	20 (14)
Male to female ratio	13:7
Diagnosed age (years) (median)	2–34 (16)
Family history	5/14
**Kidney disorder**	
Kidney hypodysplasia or unknown cause of kidney dysfunction	17
Ureterocele, double renal pelvis, and ureter	1
**Extra renal features**	
Dysplastic ears	9
Hearing loss	8
Digital anomalies	8
Anorectal anomalies	5
Intellectual disability	2
Hypothyroidism	2
Short stature	2
Congenital heart disorder	1
Ocular disorder	1
**Clinical diagnoses (number of patients)**	
Typical Townes-Brocks syndrome	8 (40%)
Townes-Brocks branchiootorenal like syndrome	4 (20%)
Non-syndromic congenital anomalies of kidney and urinary tract	7 (35%)
Unknown	1 (5%)

### Genetic analysis results (Table 1, Fig. 1).

**Fig. 1:**
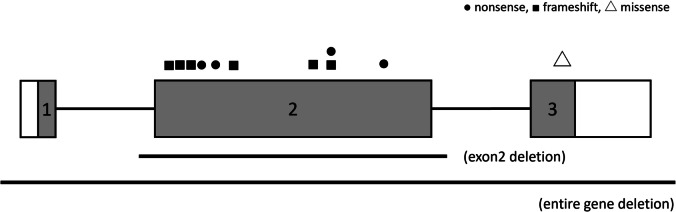
Schematic representation of the *SALL1* gene and location of the variants in the study

We identified *SALL1* variants through direct sequencing (four families), NGS (eight families), MLPA (one family), and aCGH (one family). In total, 14 types of *SALL1* aberrations were detected: frameshift (*n* = 7), nonsense (*n* = 4), missense (*n* = 1), exon 2 deletion (*n* = 1), and entire gene deletion (*n* = 1). Most pathogenic *SALL1* variants were truncating variants located in exon 2 (Fig. 1). Parent–child analyses in six families identified three patients with de novo variants (SC384, SC464, and SC624). Ten variants were not reported in the HGMD® (https://www.hgmd.cf.ac.uk/ac/index.php) or the Single Nucleotide Polymorphism Database (https://www.ncbi.nlm.nih.gov/snp/).

### Clinical phenotypes (Table 2, Fig. 2)

**Fig. 2:**
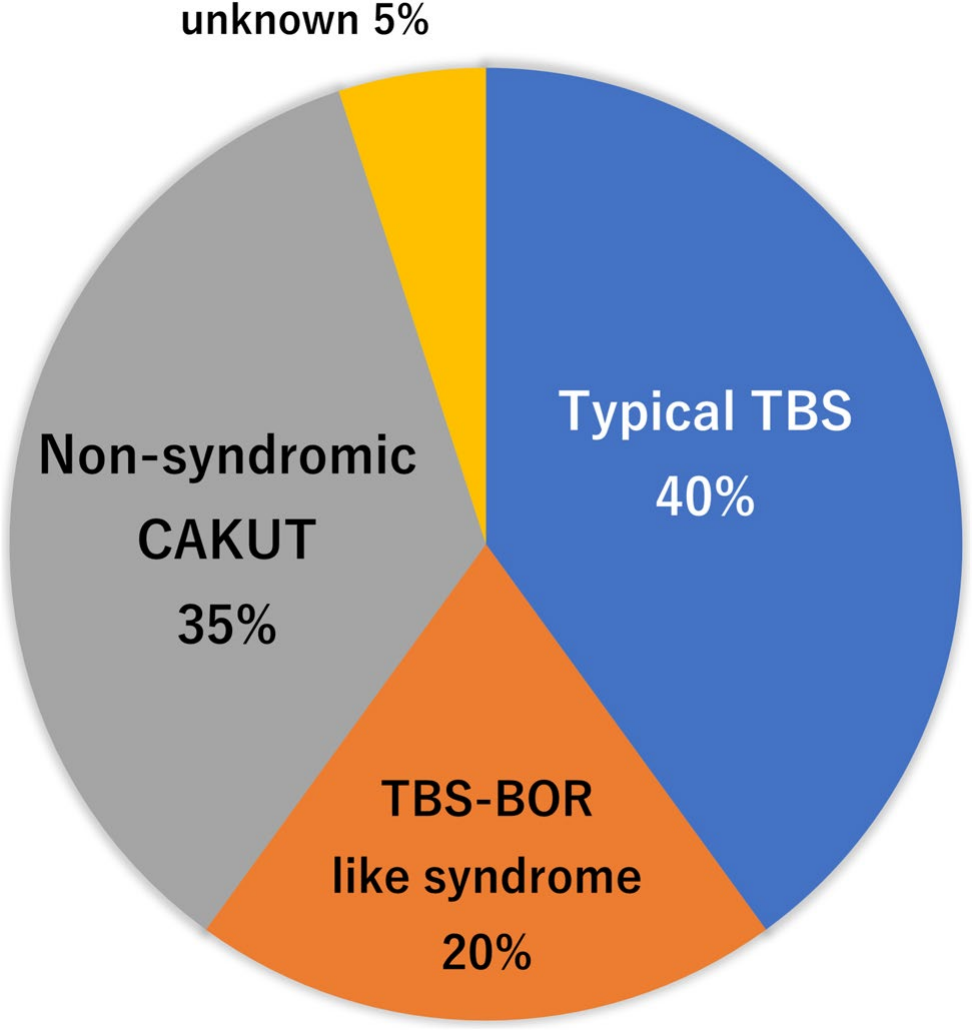
Clinical diagnosis of *SALL1*-related disorders in the study. For abbreviations, see the main text

Most patients with a pathogenic *SALL1* variant exhibited kidney involvement, with varying degrees of CKD at diagnosis. One patient (SC505) presented with ureterocele, duplex renal pelvis and ureter, while others had RHD or CKD. Extra-renal features associated with TBS1 included HL (8/20), dysplastic ears (9/20), digital anomalies (8/20), anorectal anomalies (5/20), and congenital heart disease (1/20). Additional findings included hypothyroidism (2/20), ocular features (2/20), and intellectual disability (2/20) (Table 2). The clinical diagnoses were as follows: typical TBS (*n* = 8), TBS BOR-like syndrome (*n* = 4), and non-syndromic CAKUT (*n* = 7) (Fig. 2). Clinical data were not available for one patient.

## Discussion

*SALL1* regulates the expression of numerous genes [12]. Consequently, *SALL1* abnormalities can lead to various clinical phenotypes, including TBS1. Townes first described the condition [2], and in 1998 Kohlhase et al. identified *SALL1* as the causative gene [3]. *Disheveled-binding antagonist of beta-catenin 1* (*DACT1*) has been reported as the second gene associated with TBS2 (#617,466) [13]; however, most reported cases are classified as TBS1 [14]. In our study, we performed *DACT1* analysis in 142 patients; however, no *DACT1* variants were detected.

Ten variants (excluding SC327 and SC334) were novel. Most pathogenic *SALL1* variants were truncating variants located in exon 2 (Fig. 1). One patient (SC505) had a missense variant that was absent from both the Genome Aggregation Database (https://gnomad.broadinstitute.org/) and the Japanese Multi Omics Reference Panel (https://jmorp.megabank.tohoku.ac.jp/). In silico analysis, including a CADD score of 26.5, suggested the variant was pathogenic. This patient presented solely with CAKUT. Hwang et al. reported seven patients with CAKUT who had *SALL1* missense variants [15], all located in exon 2. In contrast, the missense variant in our patient was in exon 3 and did not involve the zinc-finger domain. This variant was evaluated using the ACMG/AMP criteria (PM1, PM2, PP4, and BP1); however, the evidence is conflicting. Therefore, its pathogenicity remains uncertain.

Stein et al. reported on *SALL1*-related disorders diagnosed using a kidney gene panel [16], finding that only 14% of patients met the diagnostic criteria for TBS1. In our study, eight patients (40%) met the criteria for typical TBS1. Using the diagnostic criteria proposed by Chang et al. [17], we diagnosed four patients (20%) with TBS BOR-like syndrome, and seven (35%) with non-syndromic CAKUT. These phenotypes varied even among members of the same family, indicating intrafamilial variability (SC140 and SC587). Both families included individuals with a typical TBS1 phenotype as well as non-syndromic CAKUT. The penetrance of TBS1 is reported to be nearly 100% [4]; however, the syndrome exhibits intrafamilial heterogeneity, and its cause remains unknown. This variability highlights the broad phenotypic expressivity of TBS1.

Although *SALL1* is expressed in the cerebrum, intellectual disability and developmental delay are uncommon in *SALL1*-related disorders. We previously reported two patients with a deletion of the *SALL1* gene confirmed by aCGH [18], both of whom exhibited developmental delays. These patients also had deletions involving several other genes in addition to *SALL1*. In the current study, one patient (SC693), diagnosed by aCGH, showed no developmental delay and had a deletion region including *SALL1* as the only coding gene. Another patient (SC1136) with a *SALL1* frameshift variant presented with a TBS1 phenotype and intellectual disability. We believe that additional factors likely contribute to intellectual disability; however, the specific mechanisms remain unclear.

This study had some limitations: First, our analysis was limited to samples from patients with kidney disorders; therefore, we could not include patients with TBS1 with no kidney involvement. Yan et al. conducted a genetic analysis of 20,666 patients with HL and identified seven cases with pathogenic *SALL1* variants, one of whom had CKD accompanied by proteinuria [14]. Second, we did not perform whole-genome sequencing, which may have resulted in missing intronic pathogenic variants in *SALL1*.

## Conclusion

In conclusion, we describe the genetic and clinical characteristics of patients with *SALL1*-related disorders in a Japanese cohort. Our findings emphasize broad phenotypic variability, ranging from typical TBS to TBS BOR-like syndrome and non-syndromic CAKUT. These results highlight the importance of considering *SALL1* variants not only in patients with typical TBS features but also in those presenting with non-syndromic CAKUT. Comprehensive genetic testing, including *SALL1* analysis, is crucial for accurate diagnosis and clinical management.

## Supplementary Information

Below is the link to the electronic supplementary material.

Graphical abstract (PPTX 200 KB)

Supplementary file1 (DOCX 20.3 KB)

Supplementary file2 (DOCX 19.9 KB)

Supplementary file3 (DOCX 20.7 KB)

Supplementary file4 (DOCX 20.7 KB)

Supplementary file5 (DOCX 20.9 KB)

Supplementary file6 (DOCX 20.9 KB)

## Data Availability

The data supporting this study’s findings are available on request from the corresponding author. The data is not publicly available due to privacy restrictions.
