# Supplementary material for: Clinical characteristics of patients with SALL1-related disorder
Source: Pediatr Nephrol. 2025 Jul 14;40(11):3407–14. doi: 10.1007/s00467-025-06878-z (PMC12484339; doi:10.1007/s00467-025-06878-z)
Supplement: Supplementary file 1 — Graphical abstract (PPTX 200 KB) [file 467_2025_6878_MOESM1_ESM.pptx]

## Slide 1
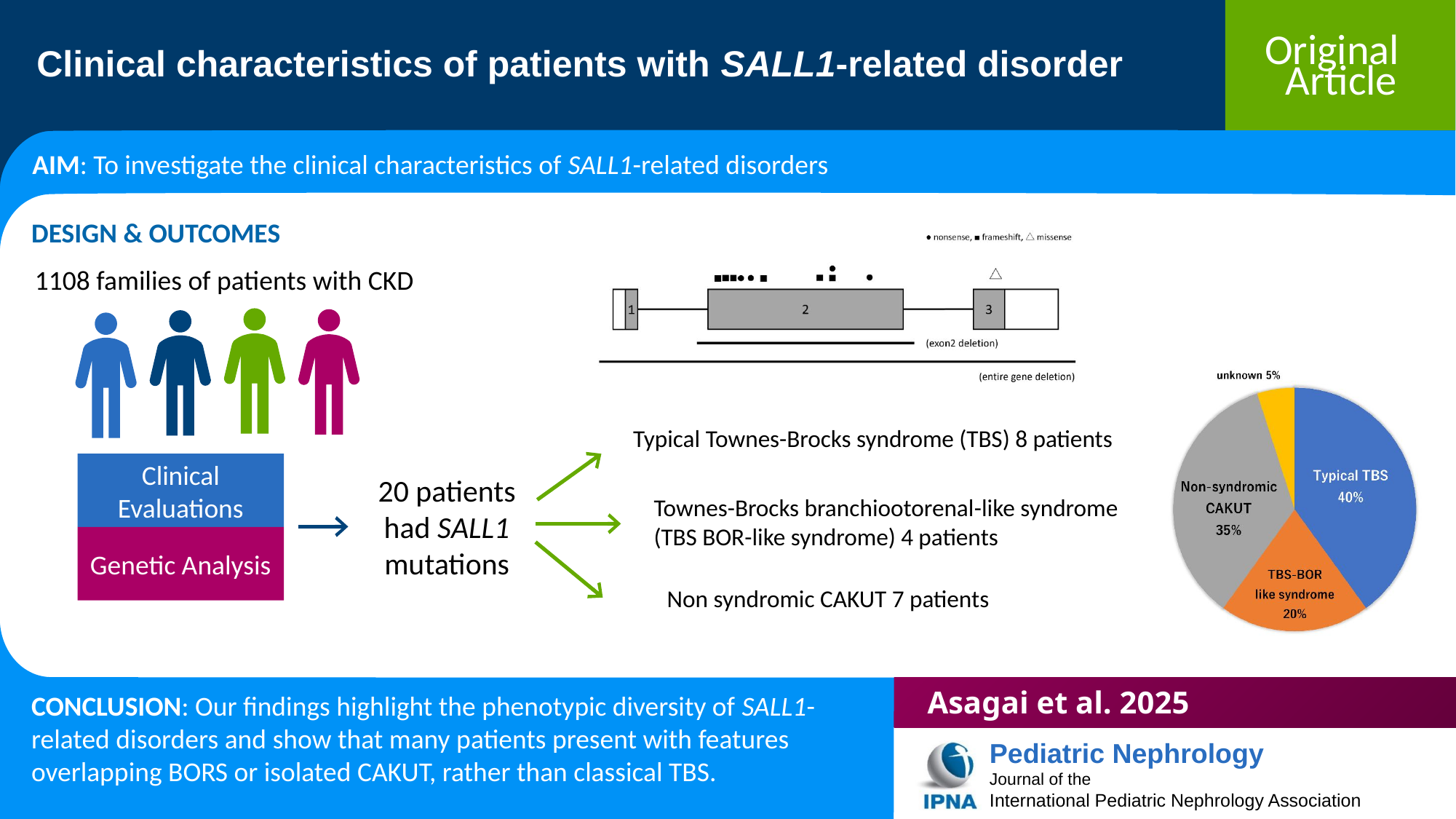

Clinical characteristics of patients with SALL1-related disorder
AIM: To investigate the clinical characteristics of SALL1-related disorders
DESIGN & OUTCOMES
1108 families of patients with CKD
Typical Townes-Brocks syndrome (TBS) 8 patients
Clinical Evaluations
20 patients had SALL1 mutations
Townes-Brocks branchiootorenal-like syndrome
(TBS BOR-like syndrome) 4 patients
Genetic Analysis
Non syndromic CAKUT 7 patients
Asagai et al. 2025
CONCLUSION: Our findings highlight the phenotypic diversity of SALL1-related disorders and show that many patients present with features overlapping BORS or isolated CAKUT, rather than classical TBS.
