# Supplementary material for: Clinical characteristics of patients with SALL1-related disorder
Source: Pediatr Nephrol. 2025 Jul 14;40(11):3407–14. doi: 10.1007/s00467-025-06878-z (PMC12484339; doi:10.1007/s00467-025-06878-z)
Supplement: Supplementary file 2 — (DOCX 20.3 KB) [file 467_2025_6878_MOESM2_ESM.docx]

Supplementary Table 1: gene list of HaloPlex HS version 4

| *ACE* | *B9D1* | *CD2AP* | *CTDNEP1* | *FOXD1* | *IFN2* | *LMNA* | *NUP107* | *RET* | *TBX1* | *TSC1* | *WNT7B* |
| --- | --- | --- | --- | --- | --- | --- | --- | --- | --- | --- | --- |
| *ACTN4* | *B9D2* | *CDC5L* | *CXCL12* | *FRAS1* | *IFT27* | *LMX1B* | *NUP133* | *ROBO2* | *TBX18* | *TSC2* | *WNT9B* |
| *ADCK4* | *BBIP1* | *CENPF* | *CXCR4* | *FREM1* | *IFT43* | *LZTFL1* | *NXF5* | *ROR1* | *TCTN2* | *TTC8* | *WNT11* |
| *AGT* | *BBS1* | *CEP41* | *DCDC2* | *FREM2* | *IFT81* | *MDM2* | *OFD1* | *ROR2* | *TCTN3* | *TTC21B* | *WT1* |
| *AGTR1* | *BBS2* | *CEP83* | *DDX59* | *FZD4* | *IFT122* | *MKKS* | *OSR1* | *RPGLIP1L* | *TMEM67* | *UMOD* | *XPNPEP3* |
| *AGTR2* | *BBS4* | *CEP104* | *DSTYK* | *FZD8* | *IFT140* | *MKS1* | *PAX2* | *SALL1* | *TMEM138* | *UPK3A* | *ZEB2* |
| *AHI1* | *BBS5* | *CEP120* | *DYNC2H1* | *GANAB* | *IFT172* | *MUC1* | *PAX8* | *SDCCAG8* | *TMEM216* | *VANGL2* | *ZNF423* |
| *ALG13* | *BBS7* | *CEP164* | *EP300* | *GATA3* | *INPP5E* | *MYO1E* | *PDE6D* | *SIX1* | *TMEM231* | *WDPCP* |  |
| *ALMS1* | *BBS10* | *CEP290* | *EYA1* | *GDNF* | *INVS* | *NEK1* | *PKD1* | *SIX2* | *TMEM237* | *WDR19* |  |
| *ANKS3* | *BBS12* | *CHD1L* | *FAT3* | *GLIS2* | *IQCB1* | *NEK8* | *PKD2* | *SIX5* | *TNXB* | *WDR34* |  |
| *ANKS6* | *BMP2* | *CHD4* | *FAT4* | *GREM1* | *ITGA8* | *NODAL* | *PKHD1* | *SLIT2* | *TP53* | *WDR35* |  |
| *ANLN* | *BMP4* | *CHRM3* | *FGF2* | *GRIP1* | *KAL1* | *NPHP1* | *PLCE1* | *SOX17* | *TRAF3IP1* | *WDR60* |  |
| *APOL1* | *BMP7* | *CITED1* | *FGF9* | *HOXA13* | *KIAA0586* | *NPHP3* | *PODXL* | *SPRY1* | *TRAP1* | *WNT4* |  |
| *ARL6* | *C5orf42* | *COQ6* | *FGF20* | *HOXD11* | *KIF14* | *NPHP4* | *PTHB1* | *SRGAP1* | *TRIM32* | *WNT5A* |  |
| *ARL13B* | *CC2D2A* | *CSPP1* | *FGFR2* | *HNF1B* | *LAMB2* | *NPHS2* | *REN* | *TBC1D1* | *TRPC6* | *WNT7A* |  |

Clinical characteristics of patients with *SALL1*-related disorder, Pediatr Nephrol, Asagai Y et al. Kobe University Graduate School of Medicine, morisada_kch@hp.pref.hyogo.jp
