# Supplementary material for: Clinical characteristics of patients with SALL1-related disorder
Source: Pediatr Nephrol. 2025 Jul 14;40(11):3407–14. doi: 10.1007/s00467-025-06878-z (PMC12484339; doi:10.1007/s00467-025-06878-z)
Supplement: Supplementary file 7 — (DOCX 20.9 KB) [file 467_2025_6878_MOESM7_ESM.docx]

Supplementary Table 6: gene list of SureSelect version 10

| *ACE* | *B9D2* | *CDC5L* | *CRB2* | *FAN1* | *GRIP1* | *IFT140* | *LRIG2* | *NPHP4* | *SALL1* | *TCTN2* | *UMOD* |
| --- | --- | --- | --- | --- | --- | --- | --- | --- | --- | --- | --- |
| *ACTG2* | *BBIP1* | *CDKN1C* | *CSPP1* | *FGF20* | *HNF1B* | *IFT172* | *LRP5* | *NRIP1* | *SARS2* | *TCTN3* | *UPK3A* |
| *AGT* | *BBS1* | *CENPF* | *DCDC2* | *FGFR1* | *HOXA11* | *INPP5E* | *LZTFL1* | *OCRL* | *SCLT1* | *TMEM67* | *VANGL2* |
| *AGTR1* | *BBS2* | *CEP41* | *DDX59* | *FGFR2* | *HOXA13* | *INTU* | *MAFB* | *OFD1* | *SDCCAG8* | *TMEM107* | *WDPCP* |
| *AGTR2* | *BBS4* | *CEP83* | *DNAJB11* | *FRAS1* | *HPRT1* | *INVS* | *MAPKBP1* | *PAX2* | *SEC61A1* | *TMEM138* | *WDR19* |
| *AHI1* | *BBS5* | *CEP104* | *DSTYK* | *FREM1* | *HPSE2* | *IQCB1* | *MKKS* | *PAX8* | *SIX1* | *TMEM216* | *WDR34* |
| *ALG8* | *BBS7* | *CEP120* | *DYNC2H1* | *FREM2* | *HYLS1* | *ITGA8* | *MKS1* | *PBX1* | *SIX2* | *TMEM231* | *WDR35* |
| *ALG9* | *BBS9* | *CEP164* | *DYNC2LI1* | *GANAB* | *INF2* | *JAG1* | *MUC1* | *PDE6D* | *SIX5* | *TMEM237* | *WDR60* |
| *ALMS1* | *BBS10* | *CEP290* | *DZIP1L* | *GATA3* | *IFT27* | *KAL1* | *MYH11* | *PIBF1* | *SON* | *TNXB* | *WNT4* |
| *ANKS6* | *BBS12* | *CFAP418* | *EP300* | *GDF11* | *IFT43* | *KIAA0556* | *MYL9* | *PKD1* | *SOX9* | *TOGARAM1* | *WT1* |
| *ARL3* | *BICC1* | *CHD1L* | *EVC* | *GDNF* | *IFT52* | *KIAA0586* | *MYLK* | *PKD2* | *SOX17* | *TRAF3IP1* | *XPNPEP3* |
| *ARL6* | *C2CD3* | *CHD4* | *EVC2* | *GFRA1* | *IFT57* | *KIAA0753* | *NEK1* | *PKHD1* | *SUFU* | *TRIM32* | *ZNF423* |
| *ARL13B* | *C5orf42* | *CHD7* | *EXOC4* | *GLIS2* | *IFT74* | *KIF7* | *NEK8* | *REN* | *TBX1* | *TSC1* |  |
| *ARMC9* | *CBWD1* | *CHRM3* | *EXOC8* | *GLIS3* | *IFT80* | *KIF14* | *NOTCH2* | *RET* | *TBX18* | *TSC2* |  |
| *ATXN10* | *CC2D2A* | *CHRNA3* | *EYA1* | *GPC3* | *IFT81* | *LMOD1* | *NPHP1* | *ROBO2* | *TCTEX1D2* | *TTC8* |  |
| *B9D1* | *CCDC28B* | *CLCN5* | *FAM149B1* | *GREB1L* | *IFT122* | *LMX1B* | *NPHP3* | *RPGRIP1L* | *TCTN1* | *TTC21B* |  |

Clinical characteristics of patients with *SALL1*-related disorder, Pediatr Nephrol, Asagai Y et al. Kobe University Graduate School of Medicine, morisada_kch@hp.pref.hyogo.jp
